# Toward community standards and software for whole-cell modeling

**DOI:** 10.1109/TBME.2016.2560762

**Published:** 2016-06-10

**Authors:** Dagmar Waltemath, Jonathan R. Karr, Frank T. Bergmann, Vijayalakshmi Chelliah, Michael Hucka, Marcus Krantz, Wolfram Liebermeister, Pedro Mendes, Chris J. Myers, Pinar Pir, Begum Alaybeyoglu, Naveen K Aranganathan, Kambiz Baghalian, Arne T. Bittig, Paulo E. Pinto Burke, Matteo Cantarelli, Yin Hoon Chew, Rafael S. Costa, Joseph Cursons, Tobias Czauderna, Arthur P. Goldberg, Harold F. Gómez, Jens Hahn, Tuure Hameri, Daniel F. Hernandez Gardiol, Denis Kazakiewicz, Ilya Kiselev, Vincent Knight-Schrijver, Christian Knüpfer, Matthias König, Daewon Lee, Audald Lloret-Villas, Nikita Mandrik, J. Kyle Medley, Bertrand Moreau, Hojjat Naderi-Meshkin, Sucheendra K. Palaniappan, Daniel Priego-Espinosa, Martin Scharm, Mahesh Sharma, Kieran Smallbone, Natalie J. Stanford, Je-Hoon Song, Tom Theile, Milenko Tokic, Namrata Tomar, Vasundra Touré, Jannis Uhlendorf, Thawfeek M Varusai, Leandro H. Watanabe, Florian Wendland, Markus Wolfien, James T. Yurkovich, Yan Zhu, Argyris Zardilis, Anna Zhukova, Falk Schreiber

**Affiliations:** Institute of Computer Science, University of Rostock, 18051 Rostock, Germany; Department of Genetics & Genomic Sciences, Icahn School of Medicine at Mount Sinai, New York, NY 10029, USA; BioQuant, University of Heidelberg, 69120 Heidelberg, Germany; European Bioinformatics Institute (EMBL-EBI), European Molecular Biology Laboratory, Cambridge CB10 1SD, UK; Department of Computing and Mathematical Sciences, California Institute of Technology, Pasadena, CA 91125, USA; Department of Biology, Humboldt University of Berlin, 10115 Berlin, Germany; Institute of Biochemistry, University Medicine Charité Berlin, 10117 Berlin, Germany; Manchester Institute of Biotechnology and the School of Computer Science, University of Manchester, Manchester M1 7DN, UK and also with the Center for Quantitative Medicine and the Department of Cell Biology, University of Connecticut Health Center, Farmington, CT 06030, USA; Department of Electrical and Computer Engineering, University of Utah, Salt Lake City, Utah 84112, USA; Gebze Technical University, Kocaeli 41400, Turkey; Department of Chemical Engineering, Boǧaziçi University, Bebek 34342, Turkey; European Bioinformatics Institute (EMBL-EBI), European Molecular Biology Laboratory, Cambridge CB10 1SD, UK; Department of Plant Sciences, University of Oxford, South Parks Road, Oxford, UK; Institute of Computer Science, University of Rostock, 18051 Rostock, Germany; Institute of Science and Technology, Federal University of São Paulo, Brazil; OpenWorm; Department of Genetics & Genomic Sciences, Icahn School of Medicine at Mount Sinai, New York, NY 10029, USA. Centre for Synthetic and Systems Biology, University of Edinburgh, Edinburgh EH9 3BF, UK; Centre of Intelligent Systems-IDMEC, Instituto Superior Técnico, University of Lisbon, 1049-001 Lisboa, Portugal; Systems Biology Laboratory, Melbourne School of Engineering, University of Melbourne, Parkville, VIC 3010, Australia, and also with the ARC Centre of Excellence in Convergent Bio-Nano Science and Technology, Melbourne School of Engineering, University of Melbourne, Parkville, VIC 3010; Faculty of Information Technology, Monash University, Clayton, VIC 3800, Australia; Department of Genetics & Genomic Sciences, Icahn School of Medicine at Mount Sinai, New York, NY 10029, USA; Department of Biosystems Science and Engineering, ETH Zürich, Basel, Switzerland; Department of Biology, Humboldt University of Berlin, 10115 Berlin, Germany; Laboratory of Computational Systems Biotechnology (LCSB), Swiss Federal Institute of Technology (EPFL), CH-1015 Lausanne, Switzerland; Laboratory of Computational Systems Biotechnology (LCSB), Swiss Federal Institute of Technology (EPFL), CH-1015 Lausanne, Switzerland; Center for Statistics, Universiteit Hasselt, Hasselt BE3500, Belgium, and also with the Center for Innovative Research, Medical University of Białystok, Białystok 15-089, Poland; Design Technological Institute of Digital Techniques, Siberian Branch of the Russian Academy of Sciences, Novosibirsk 630090, Russia; Babraham Institute, Cambridge CB22 3AT, UK; Institut für Informatik, University of Jena, 07743 Jena, Germany; Institute of Biochemistry, University Medicine Charité Berlin, 10117 Berlin, Germany. also with the Institute for Theoretical Biology, Humboldt-University Berlin, Invalidenstrae 43, 10115 Berlin, Germany; Department of Bio and Brain Engineering, Korea Advanced Institute of Science and Technology, Daejeon 305-701, Republic of Korea; European Bioinformatics Institute (EMBL-EBI), European Molecular Biology Laboratory, Cambridge CB10 1SD, UK; Sobolev Institute of Mathematics, Siberian Branch of the Russian Academy of Sciences, Novosibirsk 630090, Russia; Department of Bioengineering, University of Washington, Seattle, WA 98195, USA; CoSMo Company, Lyon, France; Stem Cell and Regenerative Medicine Research Department, Iranian Academic Center for Education, Culture Research (ACECR), Khorasan Razavi Branch, Mashhad, Iran; Rennes - Bretagne Atlantique Research Centre, Institute for Research in Computer Science and Automation, 35042 Rennes Cedex, France; Instituto de Ciencias Físicas, Universidad Nacional Autónoma de México, México; Institute of Computer Science, University of Rostock, 18051 Rostock, Germany; Department of Pharmacoinformatics, National Institute of Pharmaceutical Education and Research, Punjab 160062, India; Manchester Centre for Integrative Systems Biology, University of Manchester, Manchester M1 7DN, UK; Manchester Centre for Integrative Systems Biology, University of Manchester, Manchester M1 7DN, UK; Department of Bio and Brain Engineering, Korea Advanced Institute of Science and Technology, Daejeon 305-701, Republic of Korea; Institute of Computer Science, University of Rostock, 18051 Rostock, Germany; Laboratory of Computational Systems Biotechnology (LCSB), Swiss Federal Institute of Technology (EPFL), CH-1015 Lausanne, Switzerland. also with the Swiss Institute of Bioinformatics (SIB), CH-1015 Switzerland; Department of Dermatology, University Medicine, Friedrich-Alexander University of Erlangen-Nürnberg, Erlangen, Germany; Institute of Computer Science, University of Rostock, 18051 Rostock, Germany; Department of Biology, Humboldt University of Berlin, 10115 Berlin, Germany; Department of Systems Biology Ireland, University College Dublin, Belfield, Dublin 4, Ireland; Department of Electrical and Computer Engineering, University of Utah, Salt Lake City, Utah 84112, USA; Institute of Computer Science, University of Rostock, 18051 Rostock, Germany; Institute of Computer Science, University of Rostock, 18051 Rostock, Germany; Department of Bioengineering, University of California, San Diego, La Jolla, CA 92093, USA; Monash Institute of Pharmaceutical Sciences, Monash University, Parkville, VIC 3052, Australia; Centre for Synthetic and Systems Biology, University of Edinburgh, UK; Institut de Biochimie et Génétique Cellulaires, National Center for Scientific Research, and also with the University of Bordeaux, France, 33077 Bordeaux Cedex, France; Faculty of Information Technology, Monash University, Clayton, VIC 3800, Australia and also with the Department of Computer and Information Science, University of Konstanz, 78457 Konstanz, Germany

**Keywords:** Whole-cell modeling, Systems biology, Computational biology, Simulation, Standards, Education

## Abstract

**Objective:**

Whole-cell (WC) modeling is a promising tool for biological research, bioengineering, and medicine. However, substantial work remains to create accurate, comprehensive models of complex cells.

**Methods:**

We organized the 2015 Whole-Cell Modeling Summer School to teach WC modeling and evaluate the need for new WC modeling standards and software by recoding a recently published WC model in SBML.

**Results:**

Our analysis revealed several challenges to representing WC models using the current standards.

**Conclusion:**

We, therefore, propose several new WC modeling standards, software, and databases.

**Significance:**

We anticipate that these new standards and software will enable more comprehensive models.

**Figure:**
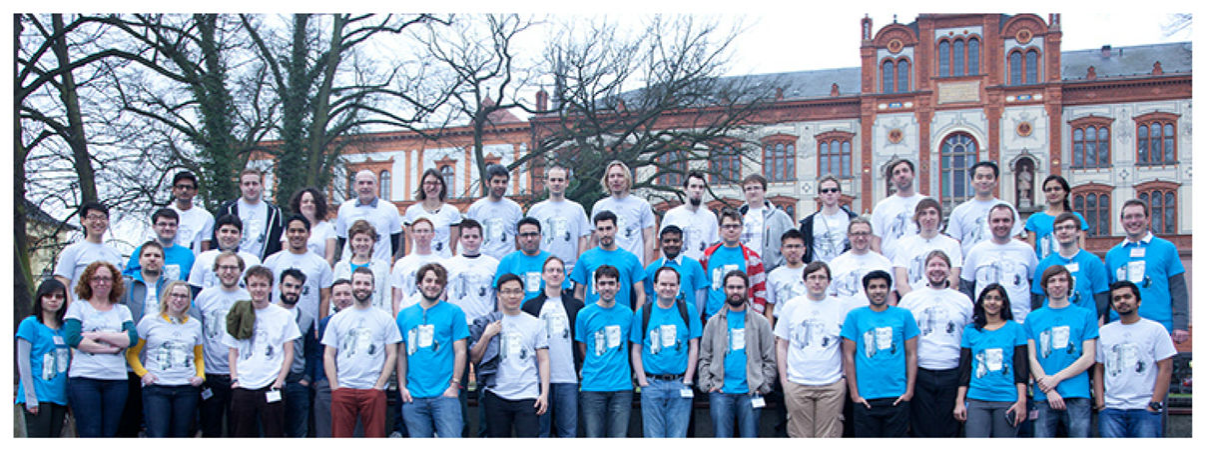
**The 2015 Whole-Cell Modeling Summer School in Rostock** included the 54 participants listed in Table SI. Photo: University of Rostock IT and Media Center.

## I. Introduction

Computational modeling is a powerful tool for biological research, bioengineering, and medicine to understand complex systems. It has been used to identify gene functions [1], engineer metabolic pathways [2], and identify drug targets [3]. Computational models also have the potential to help bioengineers design new microorganisms that can synthesize high value chemicals, sense toxins, and decontaminate waste, as well as help clinicians interpret individual ‘omics profiles and personalize medical therapy [4]. Realizing this potential requires more comprehensive models that can predict phenotype from genotype. In turn, this requires improved modeling and simulation standards and software [5–10].

Recently, Karr et al. developed the first *whole-cell* (WC) model which represents every individual gene function [11]. The model represents the life cycle of a single *Mycoplasma genitalium* bacterial cell and predicts the dynamics of every molecular species. The model is composed of 28 pathway sub-models that are represented using multiple mathematical formalisms including *stochastic simulation*, *ordinary differential equations* (ODEs), *flux balance analysis* (FBA), and *Boolean rules* (BRs). The model was implemented in MATLAB.

The *M. genitalium* model has been used to gain novel insights into non-genetic cell cycle regulation mechanisms [11], learn unknown kinetic rate parameters from phenotypic data [12], calculate the metabolic costs of synthetic circuits [13], and repurpose antibiotics [14].

Karr et al. extensively documented the model; developed the WholeCellKB [15], WholeCellSimDB [16], and Whole-CellViz [17] software tools to provide user-friendly interfaces to the model; and published the model open-source. This has enabled other researchers to reuse the model [12–14].

However, significant domain expertise is still needed to reuse the model or to develop new WC models. The multi-algorithm modeling methodology is complex. The model is difficult to understand, reuse, and extend because it is described directly in terms of its numerical simulation rather than in a software-independent format. The model code is difficult to learn and reuse because it is large, complex, and intertwined with the details of the *M. genitalium* model. The simulation code is also slow. Furthermore, the simulation code requires the proprietary MATLAB software package.

New standards and software tools are needed to help researchers build and simulate WC models. They would help researchers reuse, reproduce, and compare models, as well as share models through repositories such as BioModels [18].

Several systems biology standards have been developed by the *COmputational Modeling in BIology NEtwork* (COMBINE) [8], including the *Systems Biology Markup Language* (SBML) [19], CellML [20], the *Simulation Experiment Description Markup Language* (SED-ML) [21], and the *Systems Biology Graphical Notation* (SBGN) [22] (Table I). SBML and CellML are formats for representing mathematical models. CellML describes the mathematics whereas SBML describes biological processes. Both support several modeling formalisms including ODEs and FBA. SED-ML describes and enables researchers to reproduce computational experiments. SBGN is a visual notation for describing biological processes. However, none of these standards have been used for WC modeling.

We organized the 2015 Whole-Cell Modeling Summer School to train students in WC modeling and to evaluate the need for new WC modeling standards and software. The school focused on creating a reusable WC model by recoding the *M. genitalium* model in SBML. We focused on SBML because SBML is the most widely used systems biology standard and there was insufficient time to evaluate multiple standards. The school also aimed to improve numerous details of the model, visualize the model with SBGN, and describe model simulations with SED-ML. The latest versions of our SBML-encoded submodels and SBGN diagrams are available at https://github.com/whole-cell-tutors/wholecell/releases/tag/meeting-report.

Most importantly, the school generated extensive community discussion on how to best build and simulate WC models. This report describes the outcome of these discussions, including our recommendations for new standards and software to accelerate WC modeling. We also describe our progress toward recoding the *M. genitalium* model in SBML and the lessons that we learned about organizing research-based schools.

## II. The 2015 Whole-Cell Modeling Summer School

The school was held March 9–13, 2015, at the University of Rostock, Germany. It was organized by D. Waltemath and F. Schreiber and funded by the Volkswagen Foundation. 43 students and nine instructors participated in the school. A follow up meeting involving 15 of the original and six additional participants was held October 10–11, 2015, at the University of Utah, USA. All of the materials for the school are available at http://sites.google.com/site/vwwholecellsummerschool.

We advertised the school through community mailing lists, conference calendars, and websites. Applicants were asked to describe their experience and interest in WC modeling. We chose 43 participants from 118 applicants based on three criteria. (1) We identified the most qualified and enthusiastic applicants. (2) We gave preference to students, female applicants, and applicants from developing countries. (3) We selected participants to represent a broad range of scientific disciplines. We used the same criteria to select instructors.

The school began with introductory lectures on WC modeling and the existing systems biology standards by J. Karr and M. Hucka and introductory discussions on model composition, state representation, and stochastic modeling. Most of the school was devoted to active learning sessions in which the students and instructors were divided into 11 groups and challenged to use SBML to recode the *M. genitalium* model, use SBGN to visualize the model, and use SED-ML to simulate the model. Groups 1–8 encoded submodels. Group 9 developed a submodel integration scheme. Group 10 annotated and visualized the model. Group 11 helped all of the other groups understand, encode, and improve the model. Table SII lists the groups and participants of both meetings. Each day concluded with community discussions. In addition, the school included a poster session and networking activities.

The students learned about state-of-the-art WC modeling; the open challenges to building more complex models; open-source modeling software; the importance of reproducibility; and the SBML, SED-ML, and SBGN standards. The students also expanded their professional networks. Several of the students reported that the skills and knowledge they gained from the school would enhance their research.

We learned several lessons about organizing research-based schools. (1) Students enjoy working on research problems more than solving prescribed exercises. This engages students in the field, challenges them, and helps them build practical skills. (2) Research-based schools should have clear background knowledge expectations, learning objectives, and research goals. This helps students decide whether to participate, prepare, and learn efficiently. (3) Research-based schools should have a flexible schedule, multidisciplinary participants, and a high teacher-to-student ratio. This allows students to engage in impromptu discussions, draw on multiple perspectives, and get feedback and iterate quickly.

## III. Toward an improved SBML-encoded WC model

In addition to teaching students about WC modeling and the systems biology standards, the school aimed to improve the *M. genitalium* model and to encode the model in SBML.

### A. Submodel encoding

**Figure 1:**
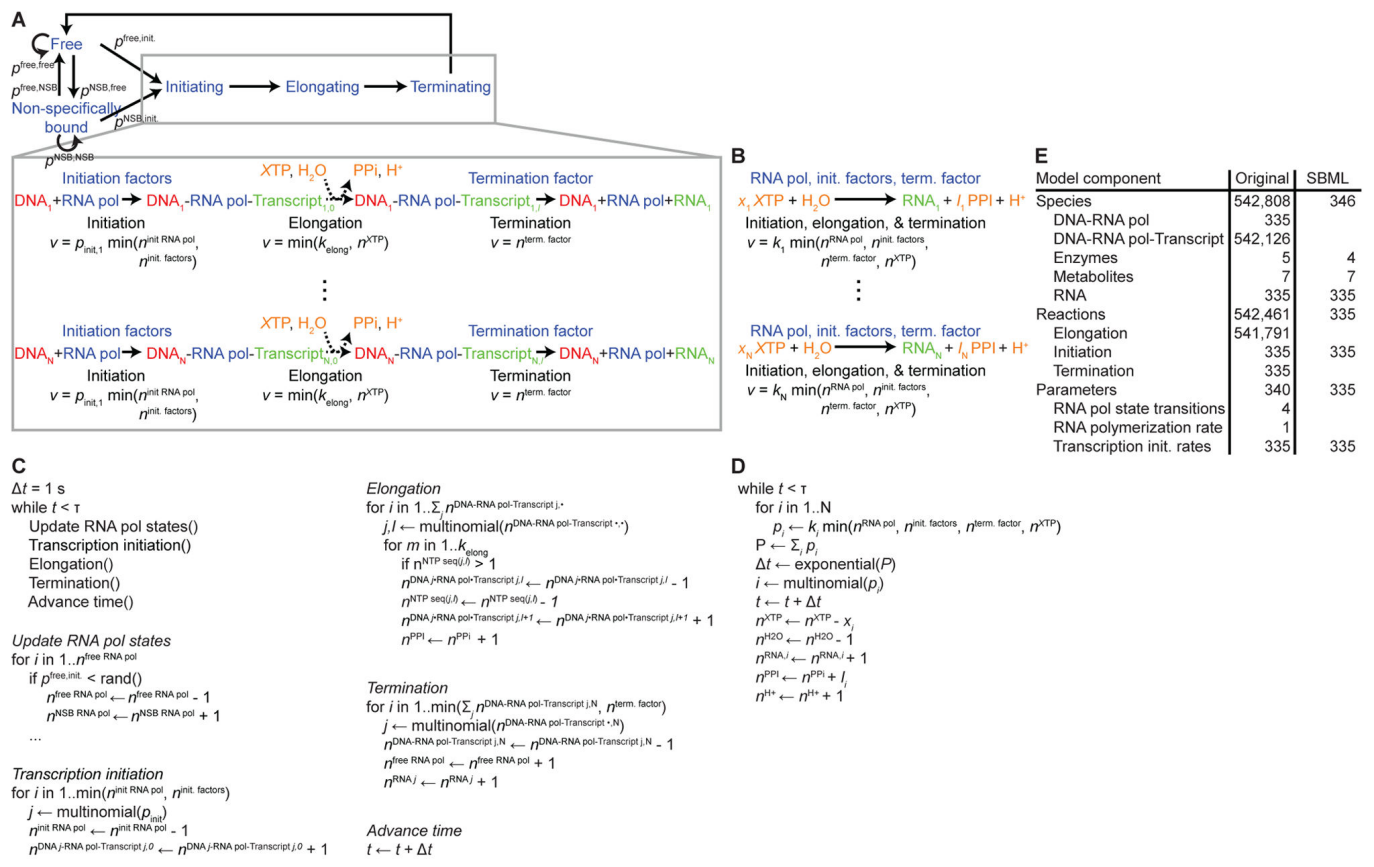
Comparison of the original and SBML transcription submodels. (A) The original transcription submodel included two sub-submodels: (1) a Markov model that describes how RNA polymerase switches among freely diffusing, non-specifically bound, and initiating states and (2) an ad hoc stochastic model that describes how RNA polymerase initiates transcription, elongates individual bases by walking along DNA, and terminates transcripts. (B) We created the SBML transcription submodel by simplifying the original submodel. The SBML submodel only represents transcription initiation, elongation, and termination; lumps the initiation, elongation, and termination of each RNA species into a single reaction; and does not explicitly represent DNA-protein binding. (C) An equivalent population-based ad hoc stochastic simulation algorithm for the original submodel. The original submodel was implemented using a more efficient particle-based algorithm. To facilitate comparison with the population-based SBML version, we have described an equivalent population-based algorithm. (D) We also improved the SBML submodel by replacing the ad hoc stochastic simulation algorithm with the Gillespie algorithm. (E) Statistics of the original and improved transcription submodels in population-based representations.

We pursued several strategies to encode submodels in SBML. Several groups encoded submodels by (1) reading the original documentation of the model, (2) drawing pathway diagrams using software tools such as CellDesigner [30] and VANTED [31], and (3) writing scripts to generate SBML from the diagrams. Other groups used model design tools such as Antimony [32], BioUML [33], COBRApy [34], COPASI [35], iBioSim [36], and libRoadRunner [37] to recode submodels based on the original documentation. A few of the groups encoded submodels by converting the MATLAB code to SBML. As an example, Fig. 1 and File S1 illustrate how we recoded the transcription submodel.

We encountered several challenges to encoding the submodels in SBML. First, understanding the submodels was time-consuming because many students were not familiar with the modeled biology, many of the submodel details are described only in the MATLAB code, and the model documentation only summarizes the model. For these reasons, J. Karr, one of the authors of the original model, helped all of the groups understand the modeled biology and mathematics. Dr. Karr also helped several groups simplify their encoding tasks by recommending that they recode only the most important model components. For example, Dr. Karr suggested that the transcription group represent the transcription of each RNA species as a single lumped reaction rather than hundreds of thousands of individual base elongation reactions. It would have been challenging to recode the model without Dr. Karr. The essentiality of Dr. Karr’s guidance underscores the need for improved WC modeling methods and standards.

Second, it was difficult to encode the original serial and randomized algorithms into SBML because SBML does not explicitly represent sequential operations and plain SBML does not support random number generation. We overcame these problems by formalizing submodels as Gillespie algorithm stochastic simulations [38].

Third, in many cases, we had to either enumerate the particle-based state representations used by the original model or approximate the original model. For example, the translation group approximated the original model by lumping all of the elongation reactions for each protein into a single reaction. The replication group used indicator variables to enumerate the particle-based chromosome representation from the original model. However, this enumerated representation requires millions of variables, which is prohibitively expensive, and makes it difficult to represent the exclusion of multiple proteins from binding the same base. Furthermore, it is impractical to edit this verbose enumerated representation.

Fourth, we had to enumerate all of the arrays used by the original model because few SBML simulators support arrays. This created verbose SBML files that are difficult to interpret and maintain and slow to simulate.

In summary, we concluded that it is currently difficult to encode WC models in SBML. WC modeling would be accelerated by expanded software support for model composition, rule-based modeling, arrays, and random number generation.

### B. Submodel improvement

We also improved several aspects of the original model. As described above, we replaced the ad hoc stochastic simulation algorithms and rate laws used by the original submodels with the Gillespie algorithm and mass action kinetics. As an example, Fig. 1 and File S1 compare the original and SBML versions of the transcription submodel. We anticipate that these changes will improve the biological accuracy of WC models. The original model used these ad hoc algorithms and rate laws to achieve sufficient performance. Going forward, a high-performance parallel simulator is needed to achieve adequate performance of the Gillespie algorithm.

### C. Model integration

The integration group created a scheme for combining the submodels. First, they defined the global species as the union of all submodel species. Second, they standardized the species names to create consistent submodel-global species interfaces.

Third, the group designed a new multi-algorithm simulation strategy to overcome the limitations of the original simulation algorithm. In particular, the group sought to correctly implement the arrow of time by integrating submodels within the same time step based on the same input state. The integration group also sought to develop an algorithm that has a variable time step that can be optimized to balance accuracy and performance. (1) The group considered sequentially integrating the submodels within each time step and setting the time step small enough that only one submodel would advance the cell state within each time step. However, this strategy is prohibitively expensive. (2) The group considered generalizing the original algorithm by dividing each of the global species pools into multiple, independent sub-species pools for each submodel; integrating the submodels in parallel; and merging the sub-species to update the global species. However, it is difficult to apply this strategy to coupled variables such as those that represent the protein occupancy of the chromosome. (3) The group decided to interpret the species changes predicted by each submodel as requests and implement a central controller that accepts or rejects these changes at the end of each time step to update the global species. This strategy is computationally efficient and generalizable.

Lastly, the group explored implementing this algorithm using both the SBML hierarchical model composition package [26] and SED-ML shared variables. The group concluded that both implementations are feasible. The group used iBioSim to test these strategies because iBioSim is one of the only SBML-compatible simulators that supports model composition.

### D. Annotation, documentation, and visualization

The documentation group was responsible for annotating the model. The group aimed to define every model element independently from external databases and to provide cross references to databases where possible to help users interpret the model. For example, they used InChI [40] to define small molecule species in terms of structures. They defined DNA, RNA, and protein species as polymers of small molecules. The group wrote scripts to identify cross references for each model entity. However, many entities are not represented by any database. The group contributed the missing metabolite structures to ChEBI [39] and concluded that the biological databases must be expanded to help aggregate data for models.

The group also helped the other groups visualize submodels by providing advice on SBGN and diagramming tools such as SBGN-ED [41], a VANTED add-on for creating, editing and validating SBGN diagrams. The main visualization problem encountered by the group was that WC models require large, intuitive diagrams that are difficult to lay out automatically.

### E. Progress and future work

We produced draft SBML and SBGN versions of the submodels. However, significant work remains to combine, identify, and verify the submodels. Using the lessons learned, a subgroup of the participants are continuing to recode the sub-models and integrate the submodels into a single model. We expect that the final model will be more scalable, extensible, and easy to use than the original model. We also plan to build an SBML-compatible multi-algorithm simulator by expanding analysis tools, such as iBioSim and BioUML.

After recoding the model, we plan to identify and validate the new model. We will validate the model in two steps. (1) We will use the experimental data that was used to validate the original model. (2) To more rigorously validate the new model, we will compare the model to newly published single-gene deletion strain growth rates [12] that were not available when the original model was developed.

We aim to publish the SBML-encoded model to BioModels, along with SED-ML tests, SBGN diagrams, and textual documentation. Publication in BioModels will make the model searchable, retrievable, and reusable. We believe this valuable community resource will demonstrate how to describe WC models in standard formats, and it will help other researchers build upon the model.

## IV. Toward SBML-, SED-ML-, and SBGN-based standards for WC modeling

The school was the first attempt to encode a WC model using standards. Thus, we were not surprised to learn that the current standards and community software do not easily support WC modeling. Importantly, the school generated ideas for new WC modeling standards and software that will enable researchers to build vastly more comprehensive models.

### A. New standards

Two new standards are needed to facilitate WC modeling. A new SBML package should be created to support DNA, RNA, and protein sequence-based reaction patterns. This would enable researchers to easily model sequence-dependent reactions such as the methylation or protein binding of specific DNA motifs. This package would also help integrate genomics and bioinformatics with systems modeling.

SBGN must also be expanded to support (1) hybrid diagrams that contain Process Description, Entity Relationship, and Activity Flow elements and (2) visualizations at multiple levels of granularity.

### B. New software tools and databases

Several new software tools and databases are needed to accelerate WC modeling (Table II). A high-performance simulator must be developed. This simulator should be parallelized to enable the simulation of vastly larger models that require more computing and memory than are available on a single machine. This requires research to determine how to concurrently integrate mathematically heterogeneous submodels that share state. The simulator should leverage recent advances in parallel discrete event simulation [42].

The simulator must also implement the SBML Multistate and Multicomponent Species package [43] to support rule-based modeling. This will enable more succinct model descriptions, making models easier to understand and edit. For example, translation could be described using a single reaction pattern parameterized by mRNA-specific translation initiation rates rather than by enumerating each individual reaction. By separating mathematical descriptions from parameter values, reaction patterns will also clarify the connection between dynamical models and their underlying data. Implementing this package would also enable modelers to efficiently simulate models with combinatorial state spaces, which, in turn, will enable the encoding of more complex models.

Ultimately, to accurately predict phenotypes, WC models must also represent spatially-dependent processes. Currently, researchers are independently pursuing WC and spatial modeling. For example, the *M. genitalium* model only represents three compartments, and the most advanced spatial models only represent individual pathways. WC and spatial modeling should be combined by adding support for the SBML Spatial Processes package [29] to the new WC simulator.

New model design software must be developed to help researchers quickly build WC models. This software should help researchers systematically build WC models from experimental data organized into pathway/genome database. In turn, this software will help researchers build bigger models.

New data curation tools are needed to aggregate data to build more comprehensive models. The software should automatically aggregate data from public databases, as well as accelerate manual curation from individual publications. This software will also make WC models more reproducible by automatically recording each data source. Natural language processing [44], crowdsourcing [45], and machine learning should also be explored to accelerate data curation.

New pathway/genome database software is needed to organize the data required to build WC models. To clarify the connection between computational models and their underlying experimental data, this software should use semantic annotations to describe how experimental data is used to build computational models.

New model parameter estimation and model verification tools are also needed to identify and verify computationally expensive WC models. To better estimate WC models, we must generalize our model reduction methods and adopt distributed numerical optimization techniques [46]. To more systematically verify WC models, we should adopt formal probabilistic verification techniques from electrical engineering [47].

New algorithms are needed to automatically create intuitive visualizations of large networks and the SBGN viewers should utilize contextual zooming to display diagrams at multiple levels of granularity.

In addition, biological databases, such as ChEBI, must be expanded to help researchers annotate WC models in terms of external entities.

### C. Systematic WC modeling pipeline

**Figure 2:**
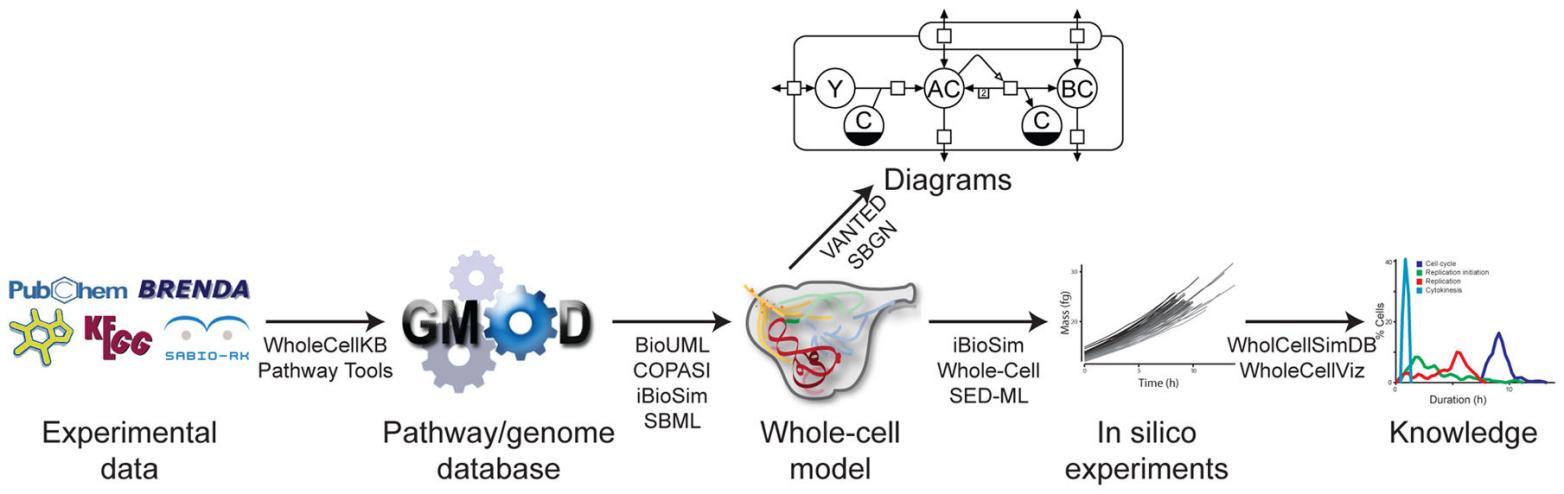
WC modeling workflow. Researchers will (1) assemble data into pathway/genome databases, (2) use these databases to construct models, (3) identify and verify models, (4) use multi-algorithm simulators to conduct *in silico* experiments, and (5) analyze these experiments to discover biology.

The new standards and software tools will enable a five step approach to WC model-driven discovery (Fig. 2). (1) Researchers will use data curation tools to aggregate heterogeneous data into pathway/genome databases. These databases will use semantic annotations to describe the connection between models and their underlying data. (2) Researchers will use design tools to build WC models from pathway/genome databases. These tools will export models to software-independent formats such as SBML. (3) Model identification and verification tools will be used to estimate parameters and test models. (4) A multi-algorithm simulator will be used to conduct *in silico* experiments. (5) Simulation databases and visualization software such as WholeCellSimDB and WholeCellViz will be used to discover new biology by visualizing and analyzing *in silico* experiments.

Together, this pipeline will enable more researchers to more easily build, manage, simulate, and reproduce WC models. These new tools will also enable researchers to build more comprehensive models of more complex eukaryotic cells. Ultimately, this will enable WC modeling to support synthetic biology and personalized medicine.

## V. Conclusion

The 2015 Whole-Cell Modeling Summer School trained young scientists in WC modeling and standards by challenging them to recode a WC model in SBML. Additional courses are needed to provide theoretical training in multi-algorithm modeling, model reduction, and parameter estimation, as well as practical training in WC model building.

We made significant strides toward recoding the model in SBML. We also improved the model by replacing the ad hoc algorithms and rate laws used by the original model with the Gillespie algorithm and mass action kinetics. We designed an improved multi-algorithm simulation meta-algorithm. Through validating the model by comparison to quantitative growth rate measurements, we anticipate that we will also discover and add several unknown parallel pathways to the model. We have produced preliminary SBML versions of all of the submodels of the *M. genitalium* model and we are working to develop a software program to simulate the combined model. We plan to publish the new SBML-encoded model to BioModels.

Most importantly, our community discussions generated clear goals for new WC modeling software and standards. We recommend that researchers develop a new SBML-compatible simulator that supports both model composition and sequence-and rule-based modeling, as well as develop new model design, parameter estimation, model testing, and visualization tools. We also recommend expanding the biological databases to facilitate model building and annotation. Furthermore, we believe that SBGN should be extended to support hybrid diagrams, advanced graph layout, and contextual zooming. Lastly, we recommend evaluating CellML as another potential WC modeling standard.

In summary, we believe that WC modeling will be an important tool for biological science, bioengineering, and medicine. Achieving this potential requires new WC modeling software and standards. In turn, this requires expanding the WC modeling field, including training young researchers.

## Figures and Tables

**Table I:** Systems biology standards and standardization efforts.

Acronym	Name	Type	Description	Ref.
CellML	CellML	Standard	Describes models in terms of mathematical relationships	20
COMBINE	COmputational Modeling in BIology NEtwork	Community	Develops computational biology standards and software	8
SBGN	Systems Biology Graphical Notation	Standard	Describes biochemical pathway diagrams	23
SBML	Systems Biology Markup Language	Standard	Describes models in terms of biochemical processes	24
SBML Arrays	SBML Package: Arrays	Standard	Describes arrays	25
SBML Comp	SBML Package: Hierarchical Model Composition	Standard	Describes how model are composed from other models	26
SBML Distrib	SBML Package: Distributions	Standard	Describes random distributions	27
SBML FBC	SBML Package: Flux Balance Constraints	Standard	Describes constraint-based models	28
SBML Multi	SBML Package: Multistate and Multicomponent Species	Standard	Supports rule-based modeling	25
SBML Spatial	SBML Package: Spatial Processes	Standard	Describes spatially-resolved models	29
SED-ML	Simulation Experiment Description Markup Language	Standard	Describes computational experiments	21

**Table II:** New standards and software needed to accelerate WC modeling.

Type	Description
Database	Expanded molecular biological databases such as ChEBI [39]
Software	Data curation tools for aggregating the data to build models
Software	Pathway/genome database to organize model training data
Standard	Sequence- and rule-based multi-algorithmic modeling language
Software	Model design tools that generate models from pathway/genome databases
Software	Distributed parameter estimation tools
Software	Frameworks for systematically verifying model
Software	High-performance, parallel, rule-based multi-algorithm simulator
Standard	Extended SBGN standard for hybrid maps containing Process Description, Entity Relationship, and Activity Flow nodes
Software	Visualization software that supports contextual zooming
